# Association between gap in spousal education and domestic violence in India and Bangladesh

**DOI:** 10.1186/1471-2458-12-467

**Published:** 2012-06-21

**Authors:** Daniel Rapp, Beate Zoch, M Mobarak H Khan, Thorsten Pollmann, Alexander Krämer

**Affiliations:** 1 Bielefeld University, School of Public Health, Department of Public Health Medicine, Universitätsstraße 25, 33615, Bielefeld, Germany

## Abstract

**Background:**

Domestic violence (DV) against women is a serious human rights abuse and well recognised global public health concern. The occurrence of DV is negatively associated with the educational level of spouses but studies dealing with educational discrepancies of spouses show contradicting results: Wives with higher education than their husbands were more likely to ever experience DV as compared to equally educated couples. The purpose of this study was to investigate the association between spousal education gap (SEG) and the prevalence and severity of DV in India and Bangladesh.

**Methods:**

Nationally representative data collected through the 2005/2006 Indian National Family Health Survey (NFHS-3) and 2007 Bangladesh Demographic and Health Survey (BDHS) were used. In total, we analysed data of 69,805 women aged 15–49 years (Bangladesh: 4,195 women, India: 65,610 women). In addition to univariate and bivariable analyses, a multinomial logistic regression model was used to quantify the association between education gap and less severe as well as severe domestic violence. Adjustment was made for age, religion, and family structure.

**Results:**

Wives with higher education than their husbands were less likely to experience less severe (OR = 0.83, 95% CI: 0.77–0.89) and severe (OR = 0.79, 95% CI: 0.72–0.87) DV as compared to equally low-educated spouses (reference group). Equally high-educated couples revealed the lowest likelihood of experiencing DV (severe violence: OR 0.43, CI 0.39–0.48; less severe violence: OR 0.59, CI 0.55–0.63). The model’s goodness of fit was low (Nagelkerke’s R^2^ = 0.152).

**Conclusions:**

Our analysis revealed no increased DV among wives with a higher educational level than their husbands. Moreover, the results point towards a decrease of severe violence with an increase in education levels among spouses. However, the model did not explain a satisfying amount of DV. Therefore, further research should be done to reveal unknown determinants so that suitable interventions to reduce DV can be developed.

## Background

Violence against women is a serious human rights violation and an important global public health problem with substantial consequences for women’s physical, mental, sexual, and reproductive health [1-6]. It is related to various adverse outcomes such as physical, sexual or mental trauma and poor health-related behaviours [7]. Women are at higher risk to experience violence from an intimate partner (also called intimate partner violence, IPV) than from any other type of perpetrator [1]. Globally, millions of women are affected by IPV or domestic violence (DV) on a daily basis [6]. Such violence is present in all countries and cuts across all kinds of social, cultural or religious groups [8]. To develop suitable interventions and give advice to policy makers it is crucial to know the determinants of DV.

Among these, the educational level of spouses is known to be negatively associated with DV [7,9]. For instance, in rural areas of Bangladesh husband’s education beyond the tenth grade was associated with a decreased risk of violence. In urban areas husband’s education beyond the sixth grade had a protective effect [10]. A study in India found an inverse relation between lifetime or recent IPV and a woman’s educational attainment [7]. Women with no formal education were 4.5 times more likely to report lifetime IPV compared with women who completed more than 12 years of education. Moreover, higher educational levels of husbands were associated with lower odds of IPV, which means that a low educated husband is a risk factor for spousal violence [10-12].

According to findings of abovementioned studies improving education is likely to be one of the key interventions to reduce DV. However, empirical data show different aspects of the association between the educational level and DV. Two studies were identified, which used educational gap between spouses (spousal educational gap, SEG) as an independent variable. One study used data from the 1998/99 Indian National Family Health Survey (NFHS) to reveal the effect of spouse’s educational discrepancy on IPV [7]. The study showed that women with higher education than their husbands were more likely to experience lifetime IPV as compared to women in marriages with no SEG (OR = 1.18; 95% CI = 1.07–1.29). However, the results of husbands with higher education than their wives were not statistically significant (OR = 1.01; 95% CI = 0.93–1.10).

Another study [11] analysed the association of partner’s educational discrepancy on the existence of IPV in a logistic regression model using cross-sectional data of Albanian women. The results showed that women with a higher educational level than their husbands were more likely to experience IPV (OR = 1) than women who were equally educated (OR = 0.40; 95% CI = 0.28–0.58; p < 0.01) or lower educated (OR = 0.21; 95% CI = 0.11–0.39; p < 0.01) than their partners. The authors assumed that physical violence is used by men to express a gender hierarchy, especially when their self-esteem is impaired by lower education than their partners. These results could lead to the assumption that educational restriction for women could reduce the number of victims of IPV.

## Methods

**Figure 1:**
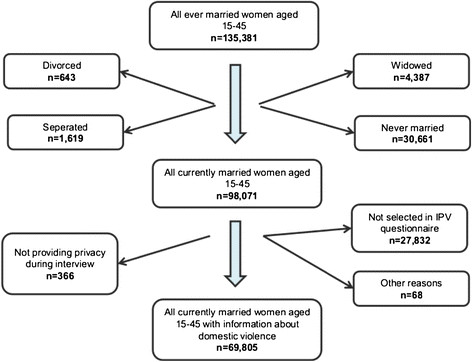
Numbers of excluded cases broken down to exclusion criteria.

We used secondary data collected through the 2005/2006 Indian National Family Health Survey (NFHS-3) and 2007 Bangladesh Demographic and Health Survey (BDHS). Both surveys drew nationally representative samples for the Indian and Bangladeshi populations by employing a multi-stage sampling design [12-14]. The information we used was related to women of reproductive ages, ranging from 15 to 49 years. The overall response rate was high in both surveys (98.4% in BDHS and 94.5% in NFHS-3). Although a total of 135,381 eligible women were indentified for the survey, our analyses were restricted to currently married women only to compare homogenous settings and to allow comparison with former studies. We excluded never married (n = 30,661), widowed (n = 4,387), divorced (n = 643) or separated women (n = 1,619). Since questions regarding DV were only asked to a randomly selected subsample, we excluded women who were not in this subsample (n = 27,832). Women associated with lack of necessary privacy during the interview (n = 366) or whereby the interview was hampered for other reasons (n = 68) were also excluded. This left a final sample of 69,805 women for analysis (Figure 1). We compared our sample with that of women who were not selected for the DV questionnaire or who were selected but could not be interviewed to reveal possible selection bias. Comparisons were made for the SEG and all factors that were considered as confounding factors in the logistic regression model. 

The sample consisted of 4,195 women from Bangladesh and 65,610 women from India. Compared with the country-specific total female population aged 15–49, the sample reflected 0.011% of women resident in Bangladesh (2005: 37,054,000 [15]) and 0.021% of women resident in India (2006: 306,468,000 [16]).

### Independent variable

SEG was ascertained by using the variables *respondent’s highest educational level* and *partner’s educational level*. Both variables measured the currently highest educational attainment and were coded equally. The educational gap was calculated by deducting the wife’s education from the husband’s education. Results with negative numbers indicated a gap in which a wife is higher educated than her husband, positive numbers revealed a gap in which a husband is higher educated than his wife. The difference equivalent to zero meant that there is no gap. In our study, the couples with equal educational level (couples with no educational gap) were furthermore divided into two subgroups by the value of their educational level. Couples with secondary or higher education and with no SEG were termed as *no gap-high education* and, equivalently, couples with no SEG and primary or no education were termed as *no gap-low education.* Thus, our main independent variable had four categories: (i) No gap-low education, (ii) wives had higher education than their husbands, (iii) husbands had higher education than their wives, and (iv) no gap-high education.

### Dependent variable

To measure spousal violence a modified version of the *Conflict Tactics Scale* (CTS) (Straus 1992) was used [12] and the respondents were asked about the situation in their current partnership. DV was ascertained by merging the variables less severe violence and severe violence into one composite dependent variable with three categories: (i) no DV experienced (ii) only less severe DV experienced and (iii) severe DV experienced. In our study, less severe violence was defined when women experienced any of the flowing options: being ‘pushed’, ‘shaken’, ‘slapped’ or even ‘kicked and punched with fist or something harmful’. Severe violence was defined when women reported any of the three options: being ‘strangled’, ‘burned’ or ‘attacked with weapons’ [12,14]. Table 1 gives an overview of the distribution of the dependent and main independent variable in the used sample in Bangladesh 2007, India 2005/06, and overall. 

**Table 1:** Overview of the distribution of the dependent and main independent variable

**Variables (n) **values	**ALL**	**BAN**	**IND**
	**% (n)**	**% (n)**	**% (n)**
**Education gap (69,298)**			
Husband higher educated	35.1 (24,306)	26.7 (1,116)	35.6 (23,190)
Wife higher educated	10.5 (7,293)	21.9 (916)	9.8 (6,377)
No gap-high education	31.5 (21,834)	19.5 (818)	32.3 (21,016)
No gap-low education	22.9 (15,865)	31.9 (1,337)	22.3 (14,528)
**Violence index (69,771)**			
No violence experienced	68.6 (47,889)	52.1 (2,185)	69.7 (45,704)
Only less severe violence experienced	21.7 (15,134)	32.1 (1,347)	21.0 (13,787)
Severe violence experienced	9.7 (9,748)	15.8 (662)	9.3 (6,086)
**Respondent’s educational level (69,799)**			
No education	38.6 (26,935)	31.9 (1,337)	39.0 (25,598)
Primary education	16.2 (11,302)	30.5 (1,280)	15.3 (10,022)
Secondary education	36.3 (25,347)	30.0 (1,258)	36.7 (24,089)
Higher	8.9 (6,215)	7.6 (318)	9.0 (5,897)

Overall (ALL; n = 69,805), in Bangladesh 2007 (BAN; n = 4,195), and in India 2005/06 (IND; n = 65,610).

We considered a number of cofactors in a multivariable regression model. The selection of cofactors was based on the literature overview as well as on the availability of variables in the dataset. Two variables (age of the spouse and the wealth index [17,18]) were considered basic cofactors. It was also assumed that the wife’s current working status [17], religion [19], attitude towards violence [18,20,21], mother’s experience of violence by her father [18,22,23], and respondent’s final say were associated with DV. The latter was measured by women’s decisive power on own health care, making large household purchases, making household purchases for daily needs and visits to family or relatives. Furthermore, rural–urban place of residence [17], relationship to head of household, and the number of household members as well as number of eligible women in the household [9] were considered. Some of the above-mentioned factors were categorized to improve the models validity. For the distribution of the cofactors included into the regression model see Table 2. 

**Table 2:** Overview of the distribution of the cofactors that were included into the regression model

**Variables (n) **values	**ALL**	**BAN**	**ND**
	**% (n)**	**% (n)**	**% (n)**
**Age (69,805)**			
Mean	31.6	30.3	31.7
≤24 yrs.	21.2 (14,770)	30.4 (1,277)	20.6 (13,493)
25-34 yrs.	42.8 (29,887)	36.8 (1,543)	43.2 (28,344)
≥35 yrs.	36.0 (25,148)	32.8 (1,375)	36.2 (23,773)
**Attitude towards domestic violence (69,666)**			
Not justified	54.7 (38,096)	68.8 (2,884)	53.8 (35,212)
Justified	45.3 (31,570)	31.2 (1,308)	46.2 (30,262)
**Religion (69,718)**			
Muslim	17.1 (11,955)	90.5 (3,795)	12.5 (8,160)
Hindu	70.6 (49,235)	8.9 (372)	74.6 (48,863)
Other	12.2 (8,528)	0.6 (27)	13.0 (8,501)
**Number of household members (69,805)**			
1-6	77.8 (54,337)	76.3 (3,202)	77.9 (51,135)
>6	22.2 (15,468)	23.7 (993)	22.1 (14,475)
**Relationship to head of household (69,805)**			
Wife	73.4 (51,223)	77.4 (3,246)	73.1 (47,977)
Daughter or daughter in law	15.9 (11,066)	8.1 (339)	16.3 (10,727)
Other	10.8 (7,516)	14.5 (610)	10.5 (6,06)
**Wealth (69,805)**			
Poorest and poorer	30.1 (21,011)	36.9 (1,550)	29.7 (19,461)
Middle	19.3 (13,473)	19.0 (798)	19.3 (12,675)
Richer and richest	50.6 (35,321)	44.0 (1,847)	51.0 (33.474)
**Final say index (69,762)**			
Wife engaged in none of the polled decisions	14.6 (10,213)	12.5 (523)	14.8 (9,690)
Wife engaged in at least one of the polled decisions	85.4 (59,549)	87.5 (3,672)	85.2 (55,877)
**Respondent’s mother experienced violence from her father (64,768)**			
No	81.6 (52,847)	74.2 (2,936)	82.1 (49,911)
Yes	18.4 (11,921)	25.8 (1,019)	17.9 (10,902)
**Type of place of residence (69,805)**			
Urban	43.5 (30,390)	37.1 (1,558)	43.9 (28,832)
Rural	56.5 (39,415)	62.9 (2,637)	56.1 (36,778)
**Respondent’s current working status (69,685)**			
No	65.1 (45,362)	70.7 (2,967)	64.7 (42,395)
Yes	34.9 (24,323)	29.3 (1,227)	35.3 (23,096)

Overall (ALL; n = 69,805), in Bangladesh 2007 (BAN; n = 4,195), and in India 2005/06 (IND; n = 65,610).

### Statistical analysis

Several statistical methods were performed using IMB SPSS Statistics 19. All calculations were done on a 5% α-level. Firstly, descriptive statistics were used to get an overview of the selected variables. Secondly, the values of Pearson’s Chi^2^-Test statistic and Spearman’s correlation coefficient (bivariable analyses) were used to check the association of selected independent variables with the dependent variable. Thirdly, we used a multivariable regression model to quantify the association between independent and dependent variables through odds ratios (ORs) and 95% confidence intervals (CI) and to adjust for cofactors. Based on the three-scaled outcome measure, an ordinal regression model could be applied. However, this procedure is critical in the present study because of its assumption of a latent metric scaled dependent variable [24]. Therefore we applied a multinomial regression model, which included the main independent variable SEG, the dependent variable DV as well as cofactors for which the association with the outcome variable was statistically significant (p < 0.05) in bivariable analyses. We also checked the models’ goodness of fit based on Nagelkerke’s R^2^ and Hosmer & Lemeshow test. We performed these calculations for the total sample as well as for the country-specific sample. To enable a comparison with the quoted studies concerning SEG, another logistic regression model was conducted, in which the exposure variable was recoded into three categories by merging *no gap-high education* and *no gap-low education*. Equally educated spouses were used as reference category. We also considered a dichotomized outcome (*no violence experienced* and *any violence experienced*).

## Results

### Descriptive results

The highest number of women in our sample belonged to the category *no education* (38.6%), followed by *secondary education* (36.3%), p*rimary education* (16.2%) and *higher education* (8.9%). A similar distribution was seen in India (*no education* 39.0%, *secondary education* 36.7%, p*rimary education* 15.3% and *higher education* 9.0%). In Bangladesh most women had *no education* (31.9%) but almost the same number had *primary* (30.5%) or *secondary education* (30.0%). 7.6% of the Bangladeshi women had *higher education*. Regarding SEG, there were more couples in Bangladesh with higher educational level of wives than their husbands compared to India (Bangladesh 21.9%, India 9.8%). A higher percentage of spouses who were equally low-educated was found in Bangladesh (Bangladesh 31.9%, India 22.3%). In contrast, there were more women in India who were less educated than their spouses (India 35.6%, Bangladesh 26.7%) and couples who were equally high-educated (India 32.3%, Bangladesh 19.5%).

**Figure 2:**
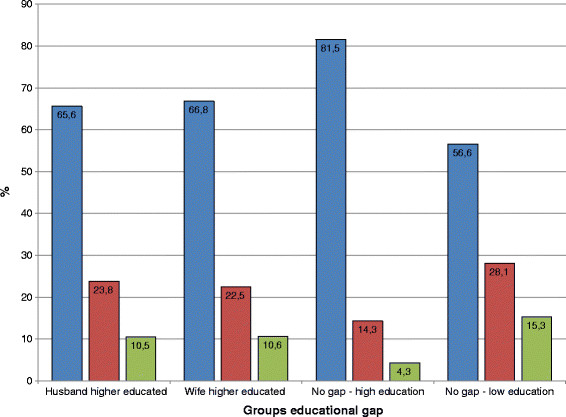
**Women's violence experienced stratified for spousal education gap value in Bangladesh 2007 (n = 4,187) and India 2005/06 (65,111) (in %), Blue – No violence experienced.** Red – Only less severe violence experienced. Green – Severe violence experienced.

Regarding DV, women experienced *no violence* in India more frequently than in Bangladesh (India 69.7%, Bangladesh 52.1%). Both types of DV were more prevalent in Bangladesh (*less severe violence* Bangladesh 32.1%, India 21.0%; *severe violence* Bangladesh 15.8%, India 9.3%). Figure 2 shows the percentage of violence by the four groups representing the SEG. Within each group of SEG, the percentage was highest for *no violence* and lowest for *severe violence*. The lowest prevalence of violence was found in the group *no gap-high education* whilst the highest prevalence of violence was found in the group *no gap-low education*. The difference between the other two groups namely *husband higher educated* and *wife higher educated* in terms of violence-level was negligible.

### Bivariable results

Some characteristics of our DV subsample were compared with the women who were not selected for the DV questionnaire or who were selected but could not be interviewed to investigate selective dropout. Except for the respondent’s educational level, all considered variables differed statistically significantly (p < 0.05, for details see Table 3). The differences in religion were particularly large with an underrepresentation of Muslims and an overrepresentation of Hindus in our subsample. This has to be considered in the interpretation of the results.

**Table 3:** Distribution of the independent variable and the cofactors in the DV subsample (n = 69,805) and the rest of the sample (n = 28,266)

	**Selected and interviewed** **(n = 69,805)**	**Not selected/not interviewed** **(n = 28,266)**	**p-value**
**Education gap**			
Husband higher educated	35.1%	36.5%	p < 0.001
Wife higher educated	10.5%	11.4%	
No gap_high education	31.5%	30.4%	
No gap_low education	22.9%	21.7%	
**Current age respondent**			
≤ 24 years	21.2%	27.8%	p < 0.001
25 to 34 years	42.8%	26.8%	
> 34 years	36.0%	45.4%	
**Highest educational level**			
No education	38.6%	36.2%	p < 0.001
Primary	16.2%	18.7%	
Secondary	36.3%	36.6%	
Higher	8.9%	8.5%	
**Wealth index**			
Poorest and poorer	30.1%	24.7%	p < 0.001
Middle	19.3%	18.7%	
Richer and richest	50.6%	56.6%	
**Number of household members (binary)**			
1–6	77.8%	44.7%	p < 0.001
>6	22.2%	55.3%	
**Religion**			
Muslim	17.1%	30.8%	p < 0.001
Hindu	70.6%	60.4%	
Other	12.2%	8.8%	
**Relationship to head of household**			
Wife	73.4%	51.6%	p < 0.001
Daughter in law	19.7%	36.6%	
Other	6.9%	11.8%	
**Working status**			
No	65.1%	69.3%	p < 0.001
Yes	34.9%	30.7%	
**Type of place of residence**			
Urban	43.5%	41.7%	p < 0.001
Rural	56.5%	58.3%	

p-values of Pearson’s Chi^2^-Test.

Before including the considered cofactors in the multivariable analysis, their associations with DV were explored by bivariable analyses and tested using Pearson’s Chi^2^ test and Spearman’s Correlation. Except for final say, all variables were statistically significant (p < 0.01) including the main independent variable spousal education gap (SEG). We tested a number of cofactors for collinearity by Spearman’s correlation co-efficient. The husband’s age was found to be highly correlated with the wife’s age (r = 0.69, p < 0.01) and the number of eligible women in the household was correlated with the number of household members (r = 0.42, p < 0.01). Therefore husband’s age and number of eligible women in the household were excluded from the multivariable analyses.

### Multivariable results

We observed a trend of a decreasing likelihood to experience DV from *no gap-low education* over *husband higher educated*, *wife higher educated* to *no gap-high education*. Couples with higher educated husbands showed significantly lower likelihood of experiencing both types of violence (*less severe violence* (OR = 0.93, CI: 0.89–0.99) and *severe violence* (OR = 0.85, CI: 0.79–0.91)) than equally low educated spouses (reference group, OR = 1). Couples with higher educated wives also showed significantly lower likelihood of experiencing *less severe violence* (OR = 0.83, 95% CI: 0.77–0.89) and *severe violence* (OR = 0.79, 95% CI: 0.72–0.87) as compared to couples who were equally low educated. Among SEG couples, the likelihood to experience DV was even lower if the wife was higher educated compared to couples with higher educated husbands. Equally high educated couples revealed the lowest likelihood of experiencing DV (*severe violence*: OR 0.43, CI 0.39–0.48; *less severe violence*: OR 0.59, CI 0.55–0.63). The same trend was found in India and in Bangladesh separately. On a country level the association of SEG with DV was significant except for the association between *less severe violence* and *wife higher educated* (p = 0.31), *less severe violence* and *husband higher educated* (p = 0.14) and *severe violence* and *husband higher educated* (p = 0.05) in Bangladesh (Tables 4 and 5).

**Table 4:** **Results of the multinomial regression model displaying the relationship of spousal education gap and domestic violence (*only less severe violence*)**

	**BAN**	**IND**	**ALL**
Wife higher educated	0.900 (0.732-1.107) p = 0.318	**0.815** (0.752-0.883) p < 0.001	**0.828** (0.768-0.892) p < 0.001
Husband higher educated	0.862 (0.708-1.049) p = 0.138	**0.939** (0.888-0.993) p = 0.028	**0.933** (0.885-0.985) p = 0.012
No gap - high education	**0.481** (0.378-0.611) p < 0.001	**0.595** (0.556-0.636) p < 0.001	**0.585** (0.548-0.625) p < 0.001

Adjusted Odds Ratios (OR, **ORs with p < 0.05 in bold letters**), 95% confidence intervals (CI) and p-value, Bangladesh 2007 (BAN, n = 3,943), India 2005/06 (IND, n = 60,060), and overall (ALL, n = 64,003), reference category *no gap-low education* and *no violence experienced,*

R^2^ = 0.131 (Cox & Snell), 0.164 (Nagelkerke).

**Table 5:** **Results of the multinomial regression model displaying the relationship of spousal education gap and domestic violence (*severe violence*)**

	**BAN**	**IND**	**ALL**
Wife higher educated	**0.633** (0.485-0.828) P < 0.001	**0.834** (0.750-0.929) p < 0.001	**0.791** (0.717-0.873) p < 0.001
Husband higher educated	0.782 (0.612-1.000) p = 0.051	**0.861** (0.810-0.926) p < 0.001	**0.851** (0.793-0.912) p < 0.001
No gap - high education	**0.338** (0.249-0.475) p < 0.001	**0.445** (0.403-0.492) p < 0.001	**0.432** (0.393-0.476) p < 0.001

Adjusted Odds Ratios (OR, **ORs with p < 0.05 in bold letters**), 95% confidence intervals (CI) and p-value, in Bangladesh 2007 (BAN, n = 3,943), India 2005/06 (IND, n = 60,060), and overall (ALL, n = 64,003), reference category *no gap-low education* and *no violence experienced,*

R^2^ = 0.131 (Cox & Snell), 0.164 (Nagelkerke).

Based on the second regression model (binary logistic regression), couples with higher educated wives (OR = 1.11, 1.05–1.18) and couples with higher educated husbands (OR = 1.22, 1.12–1.27) had a higher chance to experience DV compared to equally educated couples (reference category) (Table 6).

**Table 6:** Results of the binary logistic regression model displaying the relationship of spousal education gap and domestic violence (n = 64,003)

	**Any violence experienced**
Wife higher educated	**1.113** (1.048-1.182) p < 0.001
Husband higher educated	**1.224** (1.117-1.272) p < 0.001

Adjusted Odds Ratios (OR, **ORs with p < 0.05 in bold letters**), 95% confidence intervals (CI) and p-value, reference category *no gap* and *no violence experienced,*

R^2^ = 0.107 (Cox & Snell), 0.152 (Nagelkerke). p < 0.000 (Hosmer& Lemeshow).

The regression models’ goodness of fit was determined by calculating Nagelkerke’s R^2^ and Hosmer & Lemeshow test, although the latter test was less appropriate for our large dataset since the probability of significant results increases with higher numbers. The goodness of fit was low for both regression models. Nagelkerke’s R^2^ was 0.16 for the multinomial logistic regression model and 0.15 for the binary logistic regression model. Hence, only 16% or 15% of the variance in DV can be explained by our models. Hosmer& Lemeshow test of the binary logistic regression model was significant (*X*^2^ = 118.84, p < 0.01).

## Discussion

Our study indicates that in India of 2005/06 and Bangladesh of 2007, education had a protective effect on DV irrespectively of its distribution among spouses. Couples who were equally low-educated (primary education level or less) were the most adverse groups in terms of DV. In contrast, equally high-educated couples (secondary educational level or higher) were less likely to experience DV. The likelihood for experiencing DV in couples with educational gap lies in between couples with equally high and equally low educational level. Wives with higher education than their husbands were less likely to be exposed to DV than wives whose husbands are higher educated. Furthermore, our results point towards a reverse association of the severity of violence and educational level.

Our results are inconsistent with the literature [7,11]. An adverse effect in wives with higher education than their spouses could not be verified. However, it has to be considered that different classifications were used in different studies. A crucial limitation of earlier studies is that they did not divide the category of couples with no education gap into two subgroups (no gap-low education and no gap-high education). According to our results, these two subgroups differ remarkably with respect to violence. Assuming a general protective effect of education [7,9], merging these subgroups into one group is inappropriate.

To check the consistency of our results, we employed an additional regression model where the outcome variable was dichotomized and equally educated couples were merged into one category. The results show that equally educated spouses were the least likely to experience DV. In couples with SEG and higher educated husbands, the chance of DV was higher compared to couples with higher educated wives. These findings are also not consistent with the literature [7,11]. Several factors may explain our findings. One may be the difference in data basis. Ackerson et al. (2008) [7] used data from Indian 1998/99 NFHS. Part of the disparity of the results may come from the seven-year period between those results in comparison to the ones that were found in the present study. Burazeri et al. (2005) [11] based their research on Albanian women, which constrains comparison with data from Indian subcontinent because of culture, ethnicities and belief. A second plausible cause may be the differences in coding of the variables. As there is a high number of equally educated couples with *no* or *only primary education* and low education is associated with high odds for DV, merging the groups with no SEG should lead to an overestimation of the prevalence of violence. Thus, violence should be highly present in the merged group of the second regression model. In reality, the opposite outcome occurred which makes the matched group the least violent (Table 5). This fact can be due to a very low prevalence of DV in equally high-educated couples.

Stratified analyses were made to compare the two countries. Few differences were found between the two countries. The decreasing chance of DV in higher educated couples can be regarded irrespectively of countries. The prevalence of DV is higher in Bangladesh compared to India. Except from Bangladeshi *less severe violence* group, wives who were higher educated than their husbands were less likely to experience DV. Perhaps several results were not statistically significant for Bangladesh due to lower numbers.

The prevalence of overall DV observed in our study sample was 31.4%. In Bangladesh of 2007 the prevalence was higher (47.9%) than in India of 2005/2006 (30.3%). The prevalence in Bangladesh differed from the results of surveyed Bangladeshi men in 2004 which showed that 68% of the interviewees’ wives experienced DV [6]. This might be due to underreporting of the women because of fear and shame. The results for India are more comparable to earlier results (26% in India of 2007) [23].

### Limitations

Our study has some limitations. Firstly, the presented models had a low goodness of fit. Hence, the validity of the statistical models is low and other predictors of DV that were not included have to be considered.

Secondly, there might be selective dropouts in the selection of women for the questionnaire on DV. It can be assumed that violent settings are less likely to provide the privacy which was required to perform the survey. This fact may lead to an underestimation of violence in the current sample. Some characteristics of our final sample were significantly different from the sample that was not interviewed on DV. This narrows the generalizability of our results. Furthermore, estimating DV might be biased by the exclusion of never married, divorced and separated women as DV could be a reason for separation. Our results are affected if the chance of being separated is associated with the educational level of spouses. Nevertheless, we included only currently married women because they were supposed to have a more stable commitment and separation due to DV is less likely. Moreover, recall bias might be lower in currently married than in separated or divorced women as they are reporting on their ongoing partnership.

Another limiting factor is the validity of the outcome variable as it is categorized by DHS [12,14]. Violent acts such as kicking or punching with fist or something harmful are classified as *less severe violence*. These cases may also be considered to be classified as *severe* violence. If so, these cases are missing in the *severe violence* category, leading to an underestimation of the sample’s prevalence of severe violence.

Furthermore, as part of the construction of the outcome variable, cases that reported both types of violence, were assigned to *severe violence* which might have lead to an underestimation in the *less severe violence*-group.

We used multinomial logistic regression rather than ordinal regression since an ordinal scale could not be assumed. As a result, information of the ordinal-scaled outcome variable was lost and interpretation of the severity of DV has to be done with caution.

Furthermore, there might be a social preference bias that led to underreporting of DV, although the general set-up during the survey was arranged as supportive as possible by DHS. Women who were selected for the additional questionnaire concerning DV had to provide the necessary privacy and were excluded if the arrangement was not appropriate. Nevertheless, it has to be considered that the true unknown prevalence is higher than the surveyed due to reasons of anxiety, shame, or underestimation of the respondent. Also recall-bias can be assumed because the *ever*-experienced violence by the current husband had to be reported.

Finally, all considered factors are not capable of explaining a satisfying amount of the variance of DV. Some works show further factors that might have an impact but were not surveyed in 2007 BDHS or 2005/06 NFHS. Those factors may be alcohol or drug abuse [18,21,22,25], poor life satisfaction and well-being [21], psychiatric and psychological dysfunctions [21] as well as possible genetic dispositions.

## Conclusions

When studying the association between SEG and DV, equally-high and equally-low educated couples have to be distinguished. The odds for DV among spouses with educational gap lie in between the two extreme values of spouses, who are equally-low or equally-high educated. A dose-effect relationship was observed with the risk of DV declining with an increasing level of spouses’ education. Further investigation may focus on the relationship between SEG and DV with a special focus on the extent of the SEG as this issue was not investigated in the present study.

Regarding DV the difference between the two values of SEG (husband or wife is higher educated) was low. Moreover, there were no increased odds for wives who are higher educated than their husbands based on the used data. Therefore our results contradict the conclusion of earlier studies that higher education of women might be a risk factor for DV. Education seems to be an important factor in preventing DV irrespectively of sex. As far as the severity of DV is concerned, only assumptions can be made. The results point towards a decrease of severity of violence with an increasing amount of education among spouses. Hence, the above-mentioned educational restriction for women as a preventive step against DV is not supported by our results. Our study suggests that increasing the educational level in spouses of both sexes may be effective to reduce DV.

## Competing interests

The authors declare that they have no competing interests.

## Authors’ contributions

BZ and DR conceptualised the topic and performed analyses and wrote the draft article. MMHK provided data and background information about the culture of Bangladeshi and Indians. He also provided assistance to perform statistical analyses and revised the draft manuscript. TP and AK provided assistance to develop the research question and to perform the statistical analyses. All authors read and approved the final manuscript.

## Pre-publication history

The pre-publication history for this paper can be accessed here:

http://www.biomedcentral.com/1471-2458/12/467/prepub
